# The Ear

**Published:** 1877-12

**Authors:** 


*The Ear; Its Anatomy, Physiology, and Diseases.* A Practical Treatise for the use of Medical Students and Practitioners. By CHARLES H. BURNETT, A. M., M. D. Philadelphia: Henry C. Lea. 1877. Buffalo: T. H. Butler.

A full and comparatively exhaustive treatise is here presented. It is divided into two parts, the first giving the anatomy and physiology of the ear the second its diseases and their treatment. The anatomy is written up at length and will be found instructive to the student and general practitioner; It will be a great help to the study of the pathological changes in the various tissues.

The second part is sub-divided into seven sections treating of, examination of patients; diseases of the auricle; of the external auditory canal; of the membrana tympani; of the middle ear; of the internal ear and the last section relates to deaf mutes and partially deaf children. Section five,—diseases of the middle ear is presented in eight chapters and is an able digest of the topic and should be carefully read by every physician whether a specialist or general practitioner. Few diseases are so hard to diagnose or so important as this class. Many cases which with prompt and proper treatment would yield easily are allowed by negligence in the attending physician to go on to that serious result, incurable deafness.

Some such work as the present one should be in the library of every medical man, and for those wishing a modern carefully prepared treatise on the ear, no other is superior. C.

